# Influence of socio-demographic factors on distances travelled to access HIV services: enhanced surveillance of HIV patients in north west England

**DOI:** 10.1186/1471-2458-9-78

**Published:** 2009-03-06

**Authors:** Penny A Cook, Jennifer Downing, C Philip Wheater, Mark A Bellis, Karen Tocque, Qutub Syed, Penelope A Phillips-Howard

**Affiliations:** 1 Centre for Public Health, Liverpool John Moores University, Liverpool, UK; 2 Department of Environmental and Geographical Sciences, Manchester Metropolitan University, Manchester, UK; 3 Health Protection Agency North West, DBH House, Liverpool, UK

## Abstract

**Background:**

Patient choice and access to health care is compromised by many barriers including travel distance. Individuals with the human immunodeficiency virus (HIV) can seek free specialist care in Britain, without a referral, providing flexible access to care services. Willingness to travel beyond local services for preferred care has funding and service implications. Data from an enhanced HIV surveillance system were used to explore geodemographic and clinical factors associated with accessing treatment services.

**Methods:**

We extracted data on the location, type and frequency of care services utilized by HIV positive persons (n = 3983) accessing treatment in north west England between January 1^st ^2005 and June 30^th ^2006. Individuals were allocated a deprivation score and grouped by urban/rural residence, and distance to care services was calculated. Analysis identified independent predictors of distance travelled (general linear modelling) and, for those bypassing their nearest clinic, the probability of accessing a specialist service (logistic regression, SPSS ver 14). Inter-relationships between variables and distance travelled were visualised using detrended correspondence analysis (PC-ORD ver 4.1).

**Results:**

HIV infected persons travelled an average of 4.8 km (95% confidence intervals (CI) 4.6–4.9) per trip and had on average 6 visits (95% CI 5.9–6.2) annually for care. Longer trips were made by males (4.8 km vs 4.5 km), white people (6.2 km), the young (>15 years, 6.8 km) and elderly (60+ years, 6.3 km), those on multiple therapy (5.3 km vs 4.0 km), and the more affluent living in rural areas (16.1 km, P < 0.05). Half the individuals bypassed their nearest clinic to visit a more distant facility, and this was associated with being aged under 20 years, multiple therapy, being a male infected by sex between men, relative wealth, and living in rural areas (P < 0.05). Of those bypassing local facilities, poorer people were more likely to access a specialist centre but did not have as far to travel to do so (3.6 km) compared to those from less deprived areas (8.6 km).

**Conclusion:**

Distance travelled, and type of HIV services used, were associated with socioeconomic status, even after accounting for ethnicity, route of infection and age. Thus despite offering an 'equitable' service, travel costs may advantage those with higher income.

## Background

Tudor Hart formulated the inverse care law when he observed that people with the greatest healthcare needs often received the least adequate healthcare [1]. More than three decades later, those in lower socio-economic groups and minority ethnic groups continue to have reduced secondary and tertiary medical care compared with white people and the more affluent [2,3]. When a selection of such persons were presented with a theoretical clinical vignette of different health care options, however, their choices were similar to that made by more advantaged groups [2]. Long distance travel is recognised to be an important factor limiting patient choice and access [4,5]. The Patient Choice Project was established to offer patients using the UK's National Health Service more choice over where and when they receive treatment, and to reduce waiting times [6]. Thirty percent of respondents consistently chose 'current' over 'alternative' hospital care. Age, education and income had an important effect on the uptake of choice. Patient choice can override access barriers, and data from the USA revealed that more than half of patients surveyed in West Virginia bypassed local for more distant services [7]. Factors associated with bypass included age, income, and dissatisfaction with local services. Lack of specialty care, limited services, and the value of local services were most frequently mentioned.

Current UK health policy has a focus on 'patient choice' for hospitals in which they would like to be treated [8]. It is unclear whether choice of provider leads to less equity (the less advantaged may be less able to choose) or increased equity (everyone can access the same quality of service regardless of where they live). Key factors influencing a patient's willingness to travel beyond their local services for treatment include specialist care, reputation of hospital/surgeon, seriousness or urgency of condition, infrequent need of service, and being male and affluent [9]. Reluctance to travel is associated with being of black/minority ethnicity, and having responsibilities such as children [9]. However, such reviews have not considered other aspects of individual choice such as the need to preserve anonymity, as may be the case when people have stigmatised conditions such as sexually transmitted infections including HIV.

Persons infected with HIV are included among those most in need of high quality and accessible healthcare [10]. In the USA, barriers to care were identified for HIV positive patients accessing care, including the need of rural populations to travel great distances to maintain anonymity [11]. Socioeconomic factors and severity of HIV illness predicted use of outpatient care [12]. When universal transportation was made available to HIV patients for outpatient care in Colorado, distance to care no longer predicted poorer participation or retention by the least wealthy [13].

To preserve anonymity, individuals in the UK can self-refer to specialist services for treatment of sexually transmitted infections, including HIV care. HIV positive individuals thus have considerable flexibility as to where they access services, without referral, and may choose to boycott a local centre and travel a significant distance for treatment. Many, however, reside far from a treatment centre and have no choice but to travel. Moreover, the extent to which they exercise this choice is likely to depend on a number of issues, such as the severity of illness, their economic status, and their ability to travel to services. Data from HIV services, thus, provide a unique opportunity to evaluate factors affecting a person's choice of services. In addition, willingness to travel beyond local services for preferred care has funding and service implications.

The north west of England has had an enhanced surveillance system for HIV since 1996, collating information on demographics and use of health care services from genito-urinary medicine (GUM) clinics, infectious disease departments, haematology units and a number of other specialist units and clinics. The region covers some densely populated urban areas to the south (e.g. Merseyside; 2118 per km^2^), and some sparse rural areas to the north (e.g. Cumbria; 73 per km^2^), making it an interesting case study for travel to HIV services. The aim of this study was to explore socio-geographic factors that may influence patients' access to HIV treatment and care services. Distance travelled by HIV positive individuals for treatment was investigated to determine whether patients' socio-demographic backgrounds (age, ethnicity, level of deprivation), and their disease status (route of infection, use of antiretrovirals) impact on service choice. Assuming that individuals exercise choice over service provider, analyses at clinic level are used to determine how far people are prepared to travel to clinics offering a perceived quality of services.

## Methods

### Data extraction and distance calculations

The enhanced HIV surveillance system measures level of use of health services, demographics, place of residence and treatment variables. Pseudoanonymised identifiers are recorded in order to prevent double counting of individuals attending more than one treatment centre. Data are collected every six months, and include a summary of the care from each clinic. A subset of the variables from the hospital dataset is submitted to the national HIV surveillance system (survey of prevalent HIV infections diagnosed – SOPHID). Data collection and storage conform to all relevant laws and guidance relating to security and confidentiality. The ethical approval governing the maintenance and development of the surveillance system incorporate data extraction for monitoring and research purposes. For this study, data were extracted from the routinely collected data of all HIV positive persons accessing treatment in an eighteen month period (1^st ^January 2005 to 30^th ^June 2006; n = 4721). Of these, 718 (15.2%) were excluded for lack of post code information or for being resident outside the north west region, and a further 20 because they had not attended outpatient clinics (having had an episode of admission to hospital only), leaving data on 3983 individuals for analysis. Only HIV-specific care was included since the system does not collect data on other care, e.g. antenatal care.

The distance to all clinics offering HIV care in the north west was calculated by taking a straight line distance from the area of residence to the clinics (using postal code data) [14]. Analyses were carried out on the distance travelled per trip, but to calculate total travel burden the distance travelled for HIV care in a year was also calculated by multiplying the number of visits (for both day cases and outpatient episodes) to each location by the distance to that location. For total travel burden, distance travelled is presented as return journeys during a one-year period. Distances travelled were adjusted for those patients who were new to the database at some point after the first data collection period (i.e. if someone had attended only for the most recent six months their travel distance was doubled to make their yearly travel). The nearest clinic was also calculated, so that a comparison could be made between how far each person would have needed to travel to the nearest clinic and how far they actually travelled, with analyses by subgroup on these bypass distances. Clinics that did not offer general HIV care, and non-prescribing clinics, were excluded from this nearest clinic calculation.

### Socio-demographic variables

The North West Public Health Observatory provided Index of Multiple Deprivation (IMD) scores and rural/urban categories for each lower super output area (LSOA) of residence. Deprivation scores were ranked into three equally sized categories (least deprived, average, most deprived). Because 93% of people with HIV in the non-urban areas fell into the least deprived category, with the remaining 7% in the average category, those living in non-urban areas were coded as 'not urban least deprived', with a mean IMD of 15.3. Individuals from urban areas were split into the three deprivation groups: urban least deprived (IMD of less than 33), urban average (IMD between 33 and 57) and urban most deprived (IMD greater than 57).

Other socio-demographic variables of interest were gender, age, ethnicity category, and British residency. Each person's HIV status, in terms of route of infection, prescription of antiretroviral treatment, and year of treatment initiation, were accessible through the enhanced surveillance system.

### Statistical analyses

Variables of interest in individual subjects (n = 3983) monitored were: (i) per trip distance travelled in kilometres; (ii) whether or not the individual had travelled further than their closest service, (iii) for individuals (n = 1980) who travelled further than they needed to, the difference in distance between a journey to the nearest clinic and their actual travel distance, (iv) for those 1980 travelling further than they needed to, the type of service accessed (centre of excellence/specialist service or not).

Distance travelled and distance if travelling to the nearest clinic were positively skewed and were thus log transformed (natural logs) to improve normality for analysis of variance (ANOVA) and general linear modelling (GLM: an analysis of variance technique, SPSS ver 14). The transformation procedure resulted in 69.9% of data points lying within one standard deviation of the mean (compared to 68.3% for a theoretical normal distribution, and contrasts with 92.5% for the highly skewed original data). Summary data are converted back to kilometres for display, resulting in asymmetrical confidence intervals that represent the skew in the underlying data. ANOVA was carried out separately for each variable in turn, displaying the F statistic (see Additional File 1), degrees of freedom and associated probability for each test. Then, because many variables are related (e.g. route and sex – those infected with HIV through sex between men are, by definition, men) variables were combined and entered into a single GLM to identify independent predictors of distance and extra distance travelled. In the case of significant differences between groups, posthoc comparisons were used to identify where the significant differences occurred (Bonferroni multiple comparisons). Individuals were also coded as to whether they bypassed their local service and travelled further (extra travel) or not. The probability of bypassing the nearest clinic was analysed using backwards stepwise logistic regression. For those bypassing the nearest clinic, the probability of attending an excellent/specialist centre was also analysed by logistic regression. Analyses were carried out in SPSS (SPSS for Windows, Release 14.0; SPSS Inc).

In order to understand the inter-relationships between the variables and the distance travelled, a multivariate analysis technique (detrended correspondence analysis – DCA) was used to visualise the separation (or otherwise) of the individuals in two dimensions (i.e. scores along the two major axes of variation, based on all the measured variables) [15]. The use of two sets of axis scores enables a greater degree of visualisation of groupings than does the production of a single similarity matrix (e.g. as in cluster analysis). The first axis (axis 1) is the axis of greatest variation between individuals and provides a visualisation of all individuals (based on all of the measured variables) such that individuals with the most dissimilar profiles are found at the opposite extremes of the axis. Subsequent axes provide further separation by identifying the next largest axes of variation between individuals. Since successive axes exhibit a decreasing separation between individuals, it is common practice to use only two (or sometimes three) such axes. We have displayed the first two axes on a biplot (i.e. those axes that enable the maximum visualisation of variation). The axes are displayed in units of standard deviation (by convention multiplied by 100). DCA enables individuals to be arranged graphically on the basis of their similarity based on a large number of variables. Thus, this analysis allows us to visualise this complex dataset and to identify where separation between individuals lies. Subsequently, particular variables can be identified with particular groupings of individuals (we looked at each variable in turn to select the one that best described the separation between groups of individuals). In addition, the distribution of individuals along the axes can be correlated with a linear variable (in this case distance travelled). DCA was carried out using PC-ORD ver 4.1 [16].

## Results

### Distance travelled and number of visits

In a population of 3983 persons infected with HIV in north west England, the distances travelled to seek HIV care differed significantly according to socio-economic and demographic characteristics (Table 1). Individuals visited an average of 6 times (95% confidence intervals (95% CI) 5.9–6.2) and travelled an average of 4.8 km (95%CI 4.6–4.9) to their clinic. The average total return distance travelled per year for all clinic visits was 57.6 km (95% CI 55.4 – 59.5). Males travelled significantly further than females per trip (4.8 vs 4.5 km; P = 0.037), and individuals classified as white travelled further (5.1 km) than black African (4.0 km) and black Caribbean groups (3.1 km; multiple comparison P < 0.05). Those infected through blood/tissue and children infected from their mothers travelled the furthest (13.9 km and 7.2 km respectively), whilst those infected through heterosexual sex travelled the shortest distance (4.5 km; multiple comparisons P < 0.05). Although statistically significant, the magnitude of the difference between number of visits by category was small, ranging from 5 visits by individuals infected through injecting drug use to 7 visits by children infected by vertical transmission (P < 0.001).

**Table 1:** Differences between demographic and infection groups in number of visits and distance travelled for HIV treatment

	**N**	**Total no. visits**	**F**	**Per trip distance km**	**F**	**Total yearly return km**	**F**
**Sex**			2.1		4.2*		6.9**

Male	2945	6.1 (5.9, 6.2)		4.8 (4.7, 5.0)		59.2 (57.0, 62.1)	

Female	1038	5.8 (5.6, 6.1)		4.5 (4.3, 4.8)		53.1 (49.2, 57.0)	

**Age (years)**			5.7***		4.9***		9.7***

0–14	67	6.8 (5.9, 8.0)		6.8 (5.6, 8.0)		92.9 (74.4, 116.0)	

15–19	27	7.4 (5.6, 9.8)		4.5 (3.1, 6.3)		65.8 (39.9, 108.6)	

20–24	165	5.5 (4.9, 6.2)		4.3 (3.7, 5.0)		47.3 (40.1, 55.8)	

25–29	467	5.6 (5.2, 6.0)		4.2 (3.9, 4.5)		47.2 (42.5, 52.5)	

30–34	674	5.4 (5.1, 5.7)		4.2 (3.9, 4.5)		45.4 (41.0, 50.1)	

35–39	851	5.7 (5.5, 6.1)		4.8 (4.5, 5.1)		55.2 (50.9, 59.7)	

40–44	762	6.5 (6.1, 6.8)		5.0 (4.7, 5.5)		64.9 (59.5, 70.7)	

45–49	439	6.9 (6.5, 7.3)		5.1 (4.7, 5.6)		70.2 (63.2, 77.7)	

50–54	270	6.4 (5.8, 6.9)		5.1 (4.7, 5.8)		66.0 (57.8, 75.5)	

55–59	138	6.2 (5.5, 7.0)		5.6 (4.7, 6.6)		69.0 (56.3, 84.7)	

60+	123	6.7 (6.0, 7.4)		6.3 (5.3, 7.6)		84.8 (69.7, 103.2)	

**Ethnicity**			2.7**		12.0***		12.2***

White	2649	6.1 (6.0, 6.3)a		5.1 (5.0, 5.3)ae		63.7 (60.8, 66.6)a	

Black Caribbean	45	6.5 (5.2, 8.2)a		3.1 (2.3, 4.0)bc		38.9 (27.4, 55.4)abcd	

Black African	1082	5.8 (5.5, 6.0)ab		4.0 (3.7, 4.2)bd		46.0 (42.8, 49.2)cd	

Black Other	15	6.5 (4.7, 9.0)ab		3.7 (2.4, 5.5)acd		47.3 (29.3, 77.2)abcd	

Indian/Pakistani/Bangladeshi	48	5.5 (4.6, 6.6)ab		4.8 (3.7, 6.6)acd		54.1 (37.3, 78.2)bcd	

Other Asian/Oriental	46	5.5 (4.2, 7.1)ab		6.8 (5.0, 9.3)a		74.7 (48.6, 114.9)bd	

Other/Mixed	75	6.0 (5.1, 7.1)ab		5.5 (4.3, 7.1)ad		66.0 (48.9, 89.2)bcd	

Unknown	23	3.6 (2.5, 5.1)b		2.9 (1.4, 5.3)cde		20.3 (9.3, 44.4)bcd	

**Route of Infection**			5.1***		17.1***		15.2***

Sex between men	2172	6.3 (6.1, 6.5)ab		4.8 (4.7, 5.0)ab		60.2 (57.0, 63.1)a	

Injecting drug use	94	5.3 (4.5, 6.2)ab		5.1 (4.2, 6.3)abc		53.1 (41.5, 68.6)ab	

Heterosexual	1526	5.7 (5.5, 6.0)ab		4.5 (4.2, 4.7)ab		51.2 (48.0, 54.4)b	

Blood/tissue	57	5.9 (5.0, 7.0)ab		13.2 (10.1, 17.2)		156.4 (114.9, 212.4)c	

Mother to child	71	7.2 (6.2, 8.3)a		7.2 (6.1, 8.5)bc		104.0 (84.3, 128.1)c	

Other/unknown	63	4.9 (3.9, 6.1)b		5.8 (4.5, 7.2)abc		56.0 (39.6, 78.9)ab	

**Residency**			5.0***		18.5***		22.2***

UK	2997	6.1 (6.0, 6.3)a		5.1 (5.0, 5.3)		63.4 (60.8, 66.0)	

Asylum Seeker	367	5.5 (5.1, 6.0)ab		3.7 (3.4, 4.2)a		41.8 (37.0, 47.0)a	

Overseas Student	109	5.2 (4.5, 6.0)ab		3.5 (2.9, 4.2)a		35.7 (28.6, 44.4)a	

Other non-UK	244	6.4 (5.8, 6.9)ab		4.0 (3.5, 4.5)a		50.5 (43.8, 58.3)a	

Unknown	266	5.3 (4.8, 5.9)b		3.9 (3.4, 4.3)a		41.2 (35.1, 48.0)a	

**Level of antiretroviral therapy**			147.3***		28.9***		138.4***

None	1333	4.7 (4.5, 4.9)^Ŧ^		4 (3.9, 4.3)		38.3 (35.7, 40.9)^Ŧ^	

Mono^+^, dual^+^, triple	1935	6.4 (6.2, 6.6)^Ŧ^		5.1 (5.0, 5.5)a		66.0 (62.8, 69.5)^Ŧ^	

Quadruple or more	715	8.1 (7.7, 8.5)^Ŧ^		5.3 (4.8, 5.6)a		85.3 (78.9, 92.4)^Ŧ^	

**Deprivation and urban category**		1.6		198.1***		122.9***	

Not urban, least deprived	150	5.4 (4.8, 6.0)		16.1 (14.2, 18.3)T		173.5 (146.5, 205.0)T	

Urban, least deprived	1192	6.1 (5.9, 6.4)		6.6 (6.3, 6.9)T		80.8 (75.3, 86.3)T	

Urban, average deprivation	1347	6.1 (5.8, 6.3)		4.3 (4.2, 4.7)T		52.8 (49.6, 56.3)T	

Urban, most deprived	1294	5.9 (5.7, 6.2)		3.4 (3.2, 3.5)T		40.6 (38.3, 43.1)T	

**Total**	**3983**	**6.0 (5.9, 6.2)**		**4.8 (4.6, 4.9)**		**57.6 (55.4, 59.5)**	

Figures in brackets are lower and upper confidence intervals. Univariate analysis, ANOVA and means calculated on log transformed variables.

Total mileage is return mileage, and does not equate to number of visits multiplied by length of a single journey, since individuals may have used more than one treatment centre.

^+^9 individuals on dual, 1 on mono therapy.

Means in the same group with the same superscript letter do not significantly differ after post hoc analysis (Bonferroni multiple comparisons). Multiple comparisons by age are too complex to show here, but are typified by those in the youngest and oldest age groups being significantly higher than those in the 25 to 34 year age groups.

^Ŧ^all means significantly different from each other after Bonferroni multiple comparisons

*** P < 0.001, ** P < 0.01, * P < 0.05

Those taking antiretroviral drugs travelled significantly further than those not taking therapy (5.3 km per trip for those on four or more drugs and 5.2 km for those on three drugs, vs 4.0 km for those on none; multiple comparisons P < 0.05). This group also visited more frequently, with those on four or more antiretroviral drugs visiting on average 8 times a year and travelling a total of 85.3 km a year in round trips, compared with 5 visits made by those on no therapy (P < 0.001), and an annual travel burden of 38.3 km. People not yet taking therapy would be expected to visit clinics regularly for routine monitoring.

Age was a significant predictor of per trip travel, with the youngest (<15 years old) and the oldest (>59 years) age groups travelling significantly further per trip (6.8 km and 6.7 km respectively) compared with those of intermediate age (e.g. 4.2 km for those aged 30 to 34 years) (multiple comparisons P < 0.05). Individuals living in more rural areas travelled more than twice as far (16.1 km per trip) than those of equivalent affluence in urban areas (6.6 km). Individuals living in the most deprived urban areas travelled only 3.4 km per trip (all multiple comparisons P < 0.05). Those living in urban areas did not visit more frequently, and there was no difference in the number of visits by deprivation (P = 0.186). The per-trip distance travelled showed a similar pattern to the total annual distance travelled in a year (Table 1).

The independent relationships with per trip distance travelled shown in table 1 were confirmed using GLM. All the variables listed in table 1 were used in the GLM, but some were combined and categories were collapsed to achieve sufficient sample sizes in subgroups. These analyses are summarised here but not presented in a table. The analyses confirmed that distance travelled differed between the combined infection route and gender categories (F_3,3969 _= 10.3, P < 0.001). After post hoc multiple comparisons, only those in the 'other' category (including children, those infected through blood products and injecting drug users) travelled significantly further than all other categories (P < 0.05). Ethnicity and residency status were combined with the result that white UK nationals travelled significantly further than black African UK nationals and all categories of non-UK nationals (F_5,3969 _= 3.9, P = 0.002, multiple comparisons P < 0.05).

There was an overall effect of level of drug therapy (F_2,3969 _= 26.2, P < 0.001), and those taking antiretroviral therapy travelled significantly further than those not on therapy (multiple comparisons P < 0.05), although those on four or more drugs travelled no further than those taking three drugs (multiple comparisons P > 0.05). The strongest predictor of travel distance was deprivation/urban category (F_3,3969 _= 166.2, P < 0.001), confirming the findings in table 1, and with all categories differing significantly from each other (multiple comparisons P < 0.05). Age did not remain significant in the final model.

### Separation between groups of individuals

**Figure 1:**
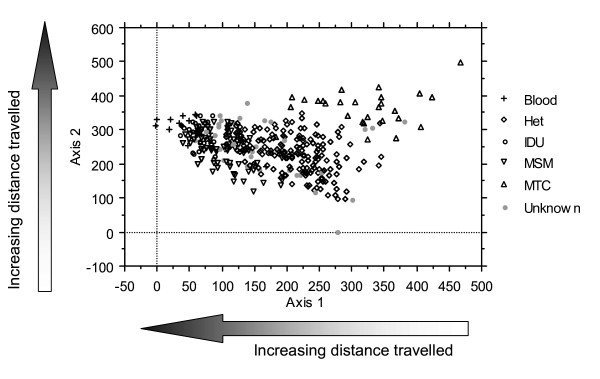
**Detrended correspondence analysis (DCA) bi-plot to visualise the relationships between demographic and infection variables (listed in table 1)**. Axes 1 and 2 are the major axes of variation between individuals based on the inclusion of all measured variables (such that those at opposite ends of an axis are the most different, hence those that are most similar can form identifiable groupings). Arrows along the axes illustrate the significant correlation between distance travelled and the axis score (Axis 1: Spearman's r_s _= -0.135, P < 0.001; Axis 2: Spearman's r_s _= 0.274, P < 0.001). The individuals are labelled by infection route (Blood = contaminated blood/blood products, Het = heterosexual, IDU = injecting drug use, MSM = men who have sex with men, MTC = mother to child).

Simultaneous expression of all variables (age, sex, ethnicity, residential status, urbanisation category, deprivation and infection route) in two dimensions (using DCA), distinguishes four main groups in the scatter plot (Figure 1). Although all variables were included in the analysis, individuals have been labelled by infection route since this variable demonstrates a clear separation between the data points. Children infected through vertical transmission lie in the top right of the graph, while those nearer the bottom left are those infected through injecting drug use and sex between men. Those to the bottom right tend to be heterosexual. Many of the variables are interrelated, for example men who have sex with men (MSM) tend to be white and older, while non-white adults tend to be heterosexual and younger (groupings not illustrated here). Distance travelled is related along both axes of Figure 1, showing that children travel further, despite being largely non-white: a category that generally travels less.

### Factors predicting extra travel

Half of the individuals (1980/3983) monitored chose to travel to a facility further than their nearest clinic (Table 2). Logistic regression revealed that extra travel was related to being aged under 20 years (P < 0.001), being on triple or more therapy (P < 0.001), being a male infected by sex between men (MSM) and relative level of deprivation by urban category, with those in rural areas and the least deprived urban areas being more likely to travel further (P < 0.001). Combining nationality and ethnicity categories did not significantly predict extra travel. This is because variation due to ethnicity and nationality was already accounted for by route of infection and deprivation (ethnic minority groups tend to be heterosexually infected and live in poorer areas).

**Table 2:** Percentage travelling further than their nearest clinic and additional distance travelled^$^

			**Predicting travelling further**			**Number of additional km**	
			
	n	df	**%**	**X^2^**	**Adj OR (95% CI)**	**Adjusted mean (95%CI)^$^**	**F**
**Age**		**3**		38.7***		**Excluded final model**	**NS**

Under 20	94		79		Reference category		

20–34	1306		47		0.341 (0.196–0.595)***		

35–49	2052		51		0.355 (0.204–0.618)***		

50+	531		51		0.321 (0.180–0.573)***		

**Level of antiretroviral therapy**		**2**		37***			6.4**

None	1333		53		Reference category	3.3 (2.9, 3.9)	

Mono/dual/triple	1935		56		1.515 (1.307–1.757)***	4.2 (3.7, 4.7)^a^	

Quadruple or more	715		44		1.658 (1.372–2.004)***	4.1 (3.5, 4.9)^a^	

**Route and sex**		**3**		68.2***			4.5**

Sex between men	2172		53		Reference category	3.1 (2.7, 3.7)^a^	

Male heterosexual	575		41		0.630 (0.503–0.788)***	4.1 (3.4, 4.9)^a, b^	

Female heterosexual	951		45		0.758 (0.608–0.945)*	4.2 (3.6, 4.8)^a, b^	

Other	285		67		1.359 (1.018–1.815)*	4.1 (3.4, 5.1)^b^	

**Deprivation and urban category**		**3**		42.3***			36***

Not urban, least deprived	150		59		1.846 (1.302–2.617)**	7.7 (5.8, 10.1)^Ŧ^	

Urban, least deprived	1192		56		1.533 (1.303–1.805)***	4.2 (3.6, 4.8)^Ŧ^	

Urban, average	1347		51		1.347 (1.152–1.574)***	3.0 (2.7, 3.4)^Ŧ^	

Urban, most deprived	1294		44		Reference category	2.3 (2.0, 2.6)^Ŧ^	

**Ethnicity and residency**		**5**		21.0**	**Excluded final model**		**2.0**

British white	2618		53			4.6 (4.1, 5.1)^a^	

British black African	419		46			4.3 (3.5, 5.2)^a^	

British other/unknown	226		52			4.4 (3.4, 5.5)^a^	

Asylum seeker	367		42			3.6 (2.9, 4.5)^a^	

Overseas student	109		44			3.5 (2.4, 5.0)^a^	

Other non national	244		48			3.1 (2.4, 4.0)^a^	

**Total**	**3983**		**50**				

Univariate Chi square analysis and multivariate logistic regression to predict extra travel. Indendent predictors of additional distance travelled determined by general linear modelling.

^$^Extra distance travelled: overall model F_13,1956 _= 16.2, P < 0.001, adjusted R2 = 0.091

^$^Adjusted means in the same group with the same letter do not significantly differ after post hoc analysis (Bonferroni multiple comparisons).

^Ŧ^all means significantly different from each other after Bonferroni multiple comparisons

*** P < 0.001, ** P < 0.01, * P < 0.05

For those persons travelling to a facility further than their nearest clinic (n = 1980), the extra distance travelled (distance to nearest clinic subtracted from actual distance travelled) was calculated (Table 2). Independent predictors of additional travel were taking complex therapy (those on quadruple therapy travelled 4.1 km compared with 3.3 km for those on no therapy, multiple comparison P < 0.05) and infection route where MSM travelled significantly less (3.1 km) than the 'other' category that included children infected from their mothers and blood/blood product recipients (4.2 km, multiple comparisons P < 0.05). There was a marginally non-significant overall difference between ethnic groups (P = 0.071), and none of the multiple comparisons achieved significance (P > 0.05). There was no difference between age or gender in terms of additional travel after controlling for the other variables. Extra travel distance correlated with level of affluence, with those in the less urban category travelling 7.7 km further than their nearest clinic, compared to 2.3 km travelled by the most deprived (urban) category (P = 0.001).

### Type of service chosen by those bypassing local services

The urban categories were more likely to bypass a local service in order to access a centre of excellence/specialist centre, and travelled less in order to do so (Table 3). While persons from the most deprived areas, who bypassed local care to access a centre of excellence, only travelled an additional 3.6 km, those from less urban and least deprived areas travelled substantially further (18.6 km and 8.6 km respectively). Compared to the less urban group, all categories of urban dwellers were more likely to access a centre of excellence/specialist centre (Table 4). The use of triple therapy also predicted visits to centres of excellence, while ethnicity and resident status did not (Table 4).

**Table 3:** Association between type of service used, distance travelled and deprivation and urbanisation category for those (n = 1980) bypassing their local service*

	**Deprivation and urban category**					
		
**Service use**		**Not urban, least deprived**	**Urban least deprived**	**Urban, average deprivation**	**Urban, most deprived**	**Total**
Centre of excellence/specialist service only^#^	% (n)	22.7 (20)	41.7 (275)	40.7 (277)	38.3 (212)	39.6 (784)
	
	Mean km (95%CI)	18.64 (11.54, 30.13)	8.55 (7.58, 9.64)	5.34 (4.74, 6.02)	3.58 (3.05, 4.2)	5.84 (5.4, 6.33)

Large centre only^$^	% (n)	18.2 (16)	17.6 (116)	13.8 (94)	15.4 (85)	15.7 (311)
	
	Mean km (95%CI)	14.52 (8.81, 23.94)	5.84 (4.88, 7)	4.6 (3.41, 6.21)	2.76 (2.09, 3.65)	4.64 (4.04, 5.34)

Smaller centre only^@^	% (n)	38.6 (34)	21.5 (142)	18.7 (127)	15.6 (86)	19.6 (389)
	
	Mean km (95%CI)	5.84 (3.69, 9.26)	4.55 (3.58, 5.79)	3.84 (3.08, 4.79)	3.96 (2.87, 5.45)	4.24 (3.69, 4.88)

Subsidiary service only^£^	% (n)	1.1 (1)	0.2 (1)	0.4 (3)	0.4 (2)	0.4 (7)
	
	Mean km (95%CI)					2.38 (0.69, 8.21)

More than one including excellence/specialist	% (n)	8.0 (7)	13.8 (91)	18.4 (125)	21.3 (118)	17.2 (341)
	
	Mean km (95%CI)	30.74 (17.56, 53.81)	9.83 (7.89, 12.25)	5.79 (4.93, 6.79)	5.45 (4.55, 6.52)	6.72 (5.96, 7.58)

More than one standard service	% (n)	11.4 (10)	5.2 (34)	7.9 (54)	9.0 (50)	7.5 (148)
	
	Mean km (95%CI)	32.97 (18.46, 58.88)	10.54 (6.66, 16.7)	6.93 (4.29, 11.2)	6.08 (3.92, 9.45)	8.13 (6.27, 10.54)

*significant: Chi square = 56.9, df = 12, p < 0.0001, excluding those attending subsidiary services only

^#^North Manchester General Infectious Disease Unit (1,500 patients), and all paediatric and haematology units

^$^Centre with over 300 HIV positive patients

^@ ^Centre with fewer than 300 HIV positive patients

^£^Non-prescribing centre

**Table 4:** Percentage of those (n = 1980) who bypass a local service to access a centre of excellence or specialist service, by demographics and infection route

			**Chi Square**			**Logistic Regression**		
	**n**	**df**	**%**	**X^2^**	**P**	**Adj odds ratio**	**(95% CI)**	**P**

**Age**				54.6	<0.001			

Under 20	74	3	87.8			Ref		0.015

20–34	603	1	47.4			0.612	(0.25, 1.502)	0.284

35–49	1038	1	59.8			0.882	(0.36, 2.159)	0.783

50+	265	1	57.7			0.772	(0.304, 1.959)	0.586

**Route and sex**				100.7	<0.001			

Sex between men	1121	3	51.3			Ref		<0.001

Male heterosexual	236	1	57.2			1.071	(0.758, 1.515)	0.697

Female heterosexual	431	1	56.1			1.032	(0.739, 1.441)	0.854

Other	192	1	90.1			8.057	(4.597, 14.122)	<0.001

**Deprivation and urban category**				28.2	<0.001			

Not urban, least deprived	88	3	30.7			Ref		<0.001

Urban, least deprived	659	1	55.5			2.703	(1.602, 4.561)	<0.001

Urban, average	680	1	59.1			3.607	(2.133, 6.100)	<0.001

Urban, most deprived	553	1	59.7			3.622	(2.123, 6.180)	<0.001

**Ethnicity and residency**				19.7	0.001			

British white	1350	5	54.4			Ref		0.032

British black African	193	1	63.7			1.22	(0.805, 1.850)	0.348

British other/unknown	118	1	57.6			0.931	(0.600, 1.443)	0.748

Asylum seeker	153	1	58.2			1.069	(0.683, 1.673)	0.771

Overseas student	48	1	81.3			3.953	(1.774, 8.808)	0.001

Other non national	118	1	61.0			1.127	(0.689, 1.842)	0.634

**Level of antiretroviral therapy**				154.4	<0.001			

None	568	2	35.4			Ref		<0.001

Mono/dual/triple	1019	1	67.3			3.56	(2.821, 4.491)	<0.001

Quad or more	393	1	60.6			2.576	(1.940, 3.420)	<0.001

**Total**	**1980**		**56.8**					

Univariate Chi square analysis and logistic regression to predict visiting a centre of excellence/specialist services.

## Discussion

Historically, UK residents with HIV have been able to choose where they access their care. As such, HIV treatment could be considered in some ways a model for examining the consequences of patient 'choice' on health services. Here we show a large variation in the amount of travel by different groups of people with HIV. Those residing in disadvantaged areas travel significantly less per trip, and are less likely to travel further than their nearest clinic, compared with those in relatively more affluent areas. This suggests that relatively wealthy individuals exercise greater choice about where they receive their HIV care. The relationship between affluence and clinic choice is complex, however, since affluent individuals who bypass local services appear, from our data, to be more likely to travel to a small centre for care than to a (large) centre of excellence or specialist centre. We suggest they travel further than they need to in order to protect their confidentiality. This appears to be particularly the case for those residing in more rural areas and replicates findings from studies in the USA [11]. We were unable to assess the relative effect of affluence on distance travelled by rural dwellers because of the negligible number of economically deprived HIV-infected patients living in rural communities.

Our findings are consistent with a review of previous studies on various conditions that determined willingness to travel was related to being male, being affluent and having more need for specialist care [9]. Of the HIV positive patients monitored here, those taking quadruple or more therapy can be assumed to require more specialist care, and these individuals travelled further per trip (4.0 km vs 5.3 km). Those with specialist needs such as children, haemophiliacs or those taking more complex drug therapy also had a higher per trip and yearly travel to access treatment. The same was true of the other variables, which demonstrated relationships between willingness (or ability) to travel and gender/infection route, affluence and complexity of treatment (level of antiretroviral therapy). In New York, admissions of patients travelling long distances outside their county was associated with younger age groups, higher illness severity and fewer same-county hospital resources [17]. Same-county admissions were associated with non-white race and lack of insurance.

In terms of addressing inequalities in access to health care, one positive finding emerged. Those in the most deprived urban areas were more likely to have accessed specialist care or care from an urban centre of excellence despite having travelled less and having been less likely to travel further than their local clinic. This reflects the placement the region's largest source of excellent/specialist care, which is located conveniently close to some of the most deprived areas. The experience of patients in north west England may not, therefore, replicate that of others living in deprived areas without similar close access to high quality services. In the USA, when transport was provided free of charge and costs reimbursed for participants needing assistance, increased distance to care did not decrease utilisation of HIV treatment programmes [13].

In this study, those with children infected with HIV suffered the greatest burden of travel when accessing specialist paediatric care. Children with HIV were more likely to travel to a clinic other than the nearest one and travelled further in a year. Distance travelled for care in chronically ill children contributes to stress in family life [18]. Added to this, two thirds of children aged less than 15 years with HIV live in the poorest fifth of the region studied. For these families, travelling long distances for hospital care is likely to be a significant financial strain.

Access to health care is complex and cannot be explained solely by distance to services. Perceptions of barriers, knowledge of services and modes of transport are all related to accessibility [9]. The analysis presented here is based on simple (straight line) distance, which is an intuitive and commonly used measure used to approximate distance along a road or public transport network [14]. For longer distances, this straight-line distance is a good representation of travel time [19], but for short urban distances, particularly in areas likely to be congested, straight-line travel is less accurate. The two alternatives, time taken to drive and use of public transport, were not used because these data were not gathered in these routinely collected surveillance data. In particular, rates of car use may be low because the majority of individuals with HIV live in the poorest parts of the study region. The analysis in this paper is limited to those individuals for whom valid postcode data were supplied. The use of postcode centroids are recognised to be less accurate in lower density areas as the same postcode is shared by people living at greater distances [20]. In our study, this limitation may mask a range of distances travelled but is unlikely to cause a systematic bias in any one direction. While the completeness of postcode data overall was good (85%), quality varied by clinic and this led to a smaller proportion of individuals from some parts of the region being included in the dataset. It would be useful to conduct a similar analysis on a national UK dataset, since individuals who travelled between regions for their HIV care were not included in this analysis. Another factor not available was distance between treatment centre and place of work; individuals with an occupation may have selected a clinic conveniently accessible from work [14].

## Conclusion

Distance travelled, and type of services used by residents of north west England with HIV, were associated with socioeconomic status, after accounting for ethnicity, route of infection and age. Thus despite offering an 'equitable' service to people living with HIV, travel costs appear to selectively advantage those with higher income. Policy makers, planners and service providers need to recognise travel remains a barrier to HIV access and care despite opportunities for 'free-choice'.

## Abbreviations

ANOVA: analysis of variance; CI: confidence intervals; DCA: detrended correspondence analysis; GLM: general linear modelling; HIV: human immunodeficiency virus; IMD: index of multiple deprivation; MSM: men who have sex with men.

## Competing interests

The authors declare that they have no competing interests.

## Authors' contributions

PAC made a substantial contribution towards conception, design and analysis of paper. JD participated in conception, design, data monitoring and preliminary analysis. CPW participated in final analysis and interpretation of data. MAB contributed to the conception and critical evaluation of content. KT contributed expertise on analysis of health inequalities and critical evaluation of content. QS participated in the design and acquisition of data. PPH participated in the interpretation of data, and to the drafting and revision of the paper, and all authors read and approved the final manuscript.

## Pre-publication history

The pre-publication history for this paper can be accessed here:



## Supplementary Material

Additional File 1 **The F statistic**. Definition of the F statistic used in Analysis of Variance and related techniques. Click here for file
