# Supplementary material for: Influence of socio-demographic factors on distances travelled to access HIV services: enhanced surveillance of HIV patients in north west England
Source: BMC Public Health. 2009 Mar 6;9:78. doi: 10.1186/1471-2458-9-78 (PMC2662835; doi:10.1186/1471-2458-9-78)
Supplement: Additional File 1 — The F statistic. Definition of the F statistic used in Analysis of Variance and related techniques. [file 1471-2458-9-78-S1.doc]

**The F statistic**

F is the theoretical probability distribution of variance ratios, which is compared to the calculated F generated in analysis of variance (ANOVA) and related techniques such as general linear modelling (GLM). In ANOVA, F is the between groups mean square divided by the within groups (or error) mean square.
